# CsBZIP40, a BZIP transcription factor in sweet orange, plays a positive regulatory role in citrus bacterial canker response and tolerance

**DOI:** 10.1371/journal.pone.0223498

**Published:** 2019-10-04

**Authors:** Qiang Li, Ruirui Jia, Wanfu Dou, Jingjing Qi, Xiujuan Qin, Yongyao Fu, Yongrui He, Shanchun Chen

**Affiliations:** 1 Citrus Research Institute, Southwest University/Chinese Academy of Agricultural Sciences, Chongqing, China; 2 Key Laboratory of Plant Hormones and Development Regulation of Chongqing, School of Life Sciences, Chongqing University, Chongqing, China; 3 School of Advanced Agriculture and Bioengineering, Yangtze Normal University, Chongqing, China; Key Laboratory of Horticultural Plant Biology (MOE), CHINA

## Abstract

Citrus bacterial canker (CBC) caused by *Xanthomonas citri* subsp. *citri* (Xcc) is a systemic bacterial disease that affects citrus plantations globally. Biotic stress in plants has been linked to a group of important transcription factors known as Basic Leucine Zippers (BZIPs). In this study, CsBZIP40 was functionally characterized by expression analysis, including induction by Xcc and hormones, subcellular localization, over-expression and RNAi silencing. CsBZIP40 belongs to group D of the CsBZIP family of transcription factors and localizes in the nucleus, potentially serving as a transcriptional regulator. In wild type (WT) plants CsBZIP40 can be induced by plant hormones in addition to infection by Xcc which has given insight into its involvement in CBC. In the present study, over-expression of CsBZIP40 conferred resistance to Xcc while its silencing led to Xcc susceptibility. Both over-expression and RNAi affected salicylic acid (SA) production and expression of the genes involved in the SA synthesis and signaling pathway, in addition to interaction of CsBZIP40 with CsNPR1, as detected by a GST pull-down assay. Taken together, the results of this study confirmed the important role of CsBZIP40 in improving resistance to citrus canker through the SA signaling pathway by the presence of NPR1 to activate PR genes. Our findings are of potential value in the breeding of tolerance to CBC in citrus fruits.

## Introduction

Citrus bacterial canker (CBC) caused by *Xanthomonas citri* subsp. *citri* (Xcc) is a systemic bacterial disease which causes severe loss in yield in most citrus-producing regions around the world. Among fruit crops, citrus is the most economically important [1]. CBC is a debilitating disease that is economically damaging at a global level. Traditionally, control of CBC has been through a classical integrated approach, including bactericides or antibiotics and eradication programs. These approaches are neither economically-viable nor friendly to the environment [2]. Improvements in citrus plants by conventional breeding strategies have been hampered by its various biological complexities [3]. Among the various breeding approaches, genetic engineering has proven to be the fastest for production of disease-resistant cultivars [4].

Potential citrus bacterial canker-related genes should be key factors in the breeding process for production of transgenic citrus varieties [4]. Several lines of transgenic citrus plants expressing genes that regulate defense mechanisms have shown significant improvements in disease resistance [5]. Genes of transcription factors (TFs) are typically candidates for molecular breeding. Introduction of TFs that regulate transcription by binding to cis-acting elements in the promoter region confers resistance against pathogens in eukaryotes [6]. Basic Leucine Zippers (BZIPs) containing TFs have a conserved basic region and a leucine zipper which binds to DNA [7–10]. The BZIP TF is a multigene family in plants. To date, BZIP TFs have been identified in numerous crops including rice, Arabidopsis, soybean, maize, grape, barley, cabbage and cucumber [11], 75 [12], 100 [9], 125 [7], 55 [13], 89 [14], 136 [15] and 64 [16]. Differences in BZIP family size confer functional variation which can be specific against diverse biotic and abiotic stress [17].

BZIP TFs are considered important regulators of many central physiological and developmental processes in plants: defense against pathogens, maturation of seeds, flowering, response to stress, *etc*. [13]. Using similarities in sequence, the 73 BZIPs in Arabidopsis can be clustered into ten BZIP sub-groups among which group D is involved in defense against pathogens [12]. Changes in their activity by modulation of the expression patterns of BZIP genes can contribute to the activation of various signaling pathways and regulatory networks of different physiological processes [18, 19]. There is no lack of functional research on BZIPs regarding biotic stress, particularly in the defense against pathogens. OBF and AtBZIP10 in *Arabidopsis thaliana* can regulate PR gene expression, HR, cell death and basal defense response [18, 20]. SlAREB1 in *Solanum lycopersicum* is involved in the response to pathogens through the ABA pathway [21]. In our previous long-term studies of citrus transcriptomes induced by Xcc, BZIP genes have always been detected among the differentially expressed genes (DEGs) (unpublished data). These results have inspired this research on the role of BZIPs in citrus bacterial canker which could potentially identify candidate genes related to infection by *Xanthomonas citri* subsp. *citri*.

In this study, we aimed to conduct comprehensive identification and functional analysis of CsBZIP40 in response to citrus bacterial canker. The involvement in CBC of the citrus bacterial canker-related CsBZIP40 gene was analyzed by its over-expression and strategies using RNAi. The molecular mechanisms of CsBZIP40-mediated CBC resistance were then analyzed in the transgenic plants.

## Materials and methods

### Plant, bacterial materials and treatments

The plant materials Calamondin (*Citrus madurensis*) and Wanjincheng (*Citrus sinensis*) used in the citrus bacterial canker and hormone assays in this study were sampled from the National Citrus Germplasm Repository, Chongqing, China (Wanjincheng: TC045; Calamondin: KPJ024). Wanjincheng was also used for gene transformation. All plants used in the study were planted at 28°C in a green house. An orange cultivation at Yunnan province, China was the source of a variant of Xcc named XccYN1 which was isolated from a sweet orange plant that naturally exhibited infection which was cultured with PYM (Peptone-yeast extract-malt extract) containing D-glucose: 1.5% (w/v) at 28°C. For analysis of expression patterns of CsBZIP40, healthy fresh leaves excised from the plants were placed in culture plates with sterile deionized water and maintained in a grow room with a 16 h light / 8 h dark photoperiod at 28°C. Xcc was cultivated at 28°C overnight, harvested at OD_600_ = 0.5 and diluted 1000-fold prior to inoculating the leaves of Wanjincheng and Calamondin. Samples were collected after 0, 6, 12, 24, 36 and 48 hours post-inoculation (hpi). To perform an exogenous hormone assay, leaf discs (diameter = 7 mm) were soaked in different hormones (10 μmol/L salicylic acid (SA), 10 μmol/L ethylene (ET), 100 μmol/L methyl jasmonic acid (MeJA)) then sampled after 0, 6, 12, 24, 36 and 48 hours post-treatment (hpt).

### Bioinformatics analysis of CsBZIPs

The Phytozome database V12 [22, 23] and CAP (Citrus Annotation Project) [24, 25] were the source of *Citrus sinensis* genome and proteome information. BZIP protein sets from *Arabidopsis thaliana* were obtained from The Arabidopsis Information Resource database (TAIR, https://www.arabidopsis.org) [26]. The CsBZIP family was annotated using a professional three-step annotation strategy [17, 27] and chromosomally visualized using Mapchart V2.1 [28]. Clustal W was used for alignment of proteins [29] and Neighbor-Joining phylogenetic trees (NJ tree) [30] constructed using Mega 7 with 1000 bootstrap replicates [31]. To reveal and visualize conserved domains, the online tool WebLogo [32] was employed. The NLS (nuclear localization signal) of CsBZIP40 was predicted using CELLO [33] combined with cNLS Mapper [34].

### Subcellular localization analysis of CsBZIP40 in onion cells

The full-length CDS of CsBZIP40 without the stop codon was amplified using primers (restriction enzymatic sites included) F-SC (CGGGGTACC ATGGCGAGTCACAGAATTGG), R-SC (TCCCCCGGG AAAGTTCGAGAAATGAT) then inserted into a pLGNe-GFP vector to construct the transient expression vector pLGNe-CsBZIP40-GFP. *Agrobacterium tumefaciens* EHA105 combined with the vector was then used to infect onion epidermal cells. The infected onion epidermal cells were used for GFP signal detection at 48 hpi. In this analysis, empty vector pLGNe-GFP was used as a control.

### Construction of over-expression and RNAi plasmids

The over-expression vector was constructed by ligation of vector pGLNe and the full-length CDS of CsBZIP40 amplified with primers F-OEc (CGCGGATCCATGGCGAGTCACAGAATTGG) and R-OEc (CGCGTCGACTCAAAAGTTCGAGAAATGAT) containing *Bam*HI and *Sal*I sites. For the RNAi vector, PUC-RNAi was integrated into a 310 bp CsBZIP40 fragment amplified by primers F-RIc (GCTCTAGAGGCGCGCCGCTGCTACTCTGGAGATGTTT) and R-RIc (CGCGGATCCATTTAAATCTGTTAATGCTGCTGCAGATT). The joint forward, intron and reverse sequence was digested by *Asc*I and *Bam*HI for insertion into pLGNe.

### Citrus transformation and characterization of transgenic lines

Stem segments of Wanjincheng were transformed by *Agrobacterium tumefaciens* EHA105 via heat shock using the method of Peng [35]. PCR and GUS assays were used to confirm the presence of transgenic genes. Primers F-OEd (TAACCAAGAAGGACCTGCTTTTGAT) and R-OEd (CTTTTCTTGTGATGGTTTTGCATCT) were used for over-expression lines and F-RId (GCAGCATTAACAGATTTAAATGTGTAA) and R-RId (TAATTGTCGGATCCAAATACCTGCAAA) for RNAi lines to detect gene integration. GUS activity in transgenic plants was evaluated using histochemistry [36]. After testing with genomic PCR and GUS assays, qRT-PCR was conducted to quantify the expression of CsBZIP40 in the positive lines with primers F-RE (AGTTCCCTTTGGGCATCTCG) and R-RE (ACCATTTGCAGATCCGTCGT). Wild type lines (WTs) were utilized as the control for PCR verification, GUS assay and qRT-PCR quantification. The qRT-PCR assay was repeated three times from which standard error was calculated.

### Assay for resistance to citrus bacterial canker

The assay for resistance to citrus bacterial canker was conducted using an *in vitro* strategy [35]. Fully matured healthy leaves from WT and transgenic lines, 6 leaves per line, were inoculated with XccYN1 *in vitro*. Four infected sites, each comprising 6 pinprick punctures (0.5 mm in diameter) per leaf were inoculated with 1 μL bacterial suspension (1 * 10^5^ CFU/mL) and incubated for 10 days. Images of the diseased regions were acquired at 10 dpi (days post-inoculation) and measured using ImageJ 2.0 software (National Institutes of Health, Bethesda, MD). The lesion area (LA) of each diseased spot and disease index (DI) observed at 10 dpi were used to evaluate resistance to Xcc. The DI was calculated using the formula reported previously [35]. The assessment of transgenic plant resistance against citrus bacterial canker were repeated three times as described above.

### GST pull-down and analysis of interacting proteins from CsBZIP40

To obtain the proteins that interacted with CsBZIP40, a GST pull-down assay was used. Total protein was extracted from the leaves of OE1. Prokaryotic expression, purification of CsBZIP40 proteins and the GST-pull down assay, together with LC/MS were performed as a service by DetaiBio Ltd. (Nanjing, China). The proteins identified using LC/MS (liquid chromatography-mass spectrometry) were annotated based on information from the CAP database and the interaction network analyzed using STRING (https://string-db.org/) with *Arabidopsis thaliana* as the reference organism.

### RNA isolation, cDNA synthesis and qRT-PCR assay

For quantitative RT-PCR, frozen tissue was ground in liquid nitrogen and then total RNA extracted from leaf samples using a Miniprep kit purchased from AidLab Ltd. (Beijing, China). The RNA was then reverse transcribed to cDNA using a kit from TaKaRa (Dalian, China). A SYBR Premix kit (Bio-Rad, USA) and the Quant Studio 7 system (Applied Biosystems, USA) were used for qRT-PCR, with actin from citrus (GenBank accession: GU911361.1) used as the internal control gene, amplified using primers F-actin (CATCCCTCAGCACCTTCC) and R-actin (CCAACCTTAGCACTTCTCC). qRT-PCR was performed by heating samples to 95°C pre-denaturation for 5 min, followed by 40 cycles at 95°C for denaturation for 10 s, and 56°C for annealing and extension for 30 s. The 20 μL reaction mixture comprised 100 ng cDNA, 0.5 μM primers and 10 μL SYBR Green PCR mix. The 2^-ΔΔCT^ method was used to evaluate relative expression levels of each gene investigated [37]. Specific primers for CsPR1, CsPR5, CsNPR1 and CsICS were designed by NCBI Primer Blast program and are listed in S1 Table. For each gene, three biological and three technical replicates were used for the qRT-PCR assay.

### Evaluation of SA concentration in transgenic plants

To confirm the induction of hormones by CsBZIP40, SA was extracted from the leaves of transgenic and WT lines for the evaluation of SA concentration. One g of fresh leaf samples was collected and ground to a fine powder, then sequentially extracted with 80% methanol overnight and centrifuged at 13,000 r/min for 10 min. The supernatant was evaporated and the residue then dissolved in 1% acetic acid. The hormones were purified using Oasis cartridges (Water, Milford, MA, USA) in accordance with the manufacturer’s instructions. The extracted hormones were dissolved in 10% methanol, then quantified using HPLC (high pressure liquid chromatography). This test was repeated three times.

### Statistical analysis

SPSS software (Version 22) was utilized for ANOVA using Fisher’s least significant difference (LSD) test, with results considered significant when P<0.05.

## Results

### Isolation and bioinformatic analysis of CsBZIP40

**Fig 1:**
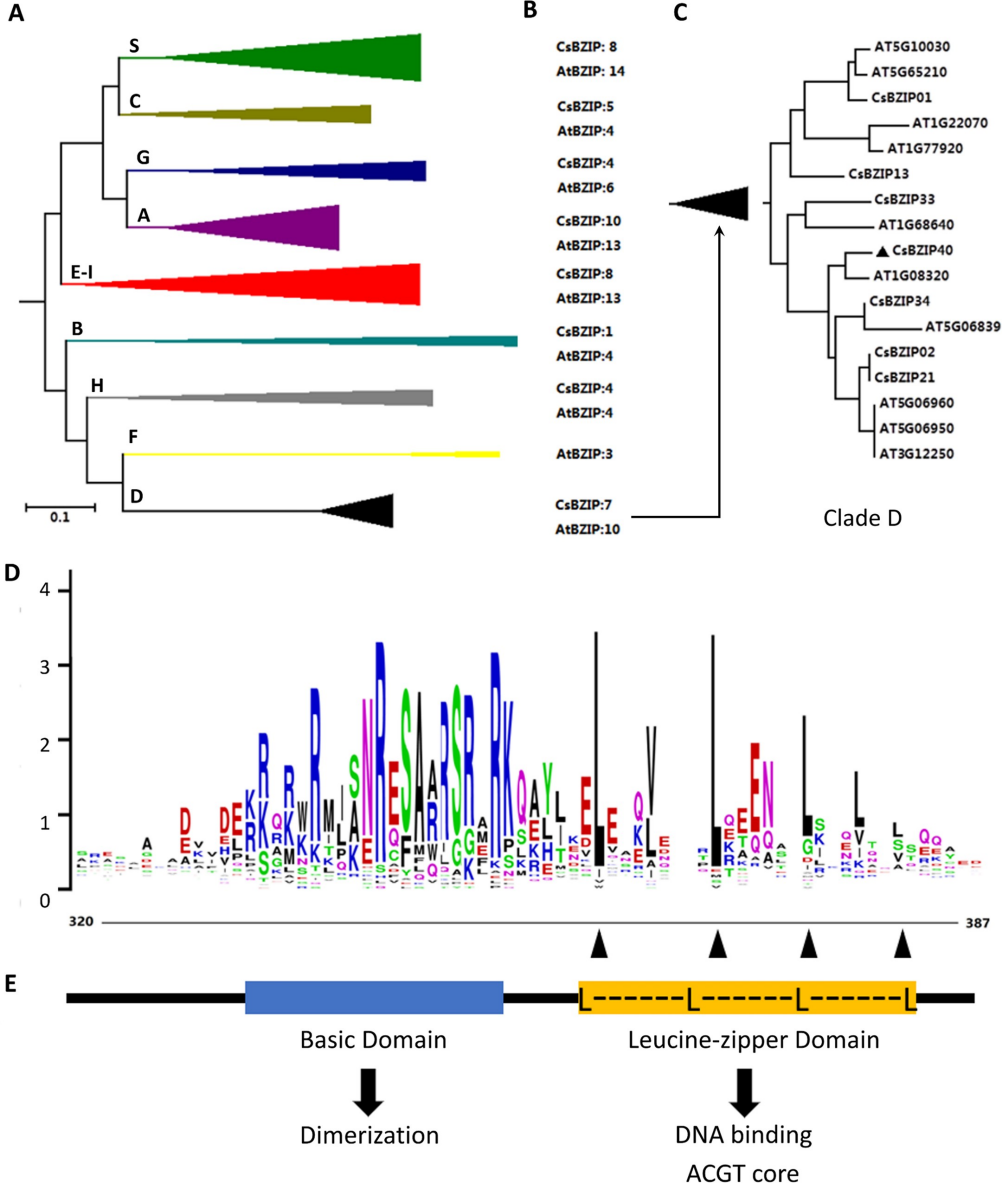
Bioinformatics analysis of CsBZIP40. **(A)** Phylogenetic relationship of BZIP transcription factors between *Citrus sinensis* and *Arabidopsis thaliana* (compressed Neighbor-Joining tree). The phylogeny was analyzed with full-length protein seqs using ClustalW and MEGA7.0. Bootstrap replicates = 1000. The letters (A-F, S) in the tree represent sub-groups. **(B)** Numbers of genes in *Citrus sinensis* and *Arabidopsis thaliana* in each group. **(C)** Expanded visualization of group D, CsBZIP40 is marked with a black diamond. **(D)** Conserved motif of CsBZIPs visualized by WebLogo. **(E)** Illustration of CsBZIP40 domains. L: Leucine.

A total of 47 potential BZIP genes were annotated from the whole genome of *Citrus sinensis* [10], based on a comprehensive annotation procedure. Each was termed ‘CsBZIP’ followed by a number representing the location and order on the chromosomes (S2 Table). Based on chromosomal localization, the 47 CsBZIPs were distributed unevenly on every chromosome, except chromosome 9 (S3 Table). The ML phylogeny of BZIPs in sweet orange and *A*. *thaliana* were constructed with full-length protein sequences of BZIPs to reveal aspects of evolution in terms of classification and relationship (S4 Table). The ML tree categorized CsBZIPs into 10 subfamilies (Fig 1A and 1B). Interestingly, CsBZIP40 was found to be an orthologous gene to AtBZIP21 in group D (At1g08320) (Fig 1C), a gene pertinent to the pathogenic defense in *Arabidopsis thaliana* [12]. CsBZIPs contain a basic domain used for protein dimerization and a following leucine zipper region containing several heptad repeats (Fig 1D). CsBZIP40 contained both classic domains which indicated BZIP identity which should bind a target with a typical ACGT core existing in various promotors (Fig 1E) [38].

### CsBZIP40 localizes to the nucleus

**Fig 2:**
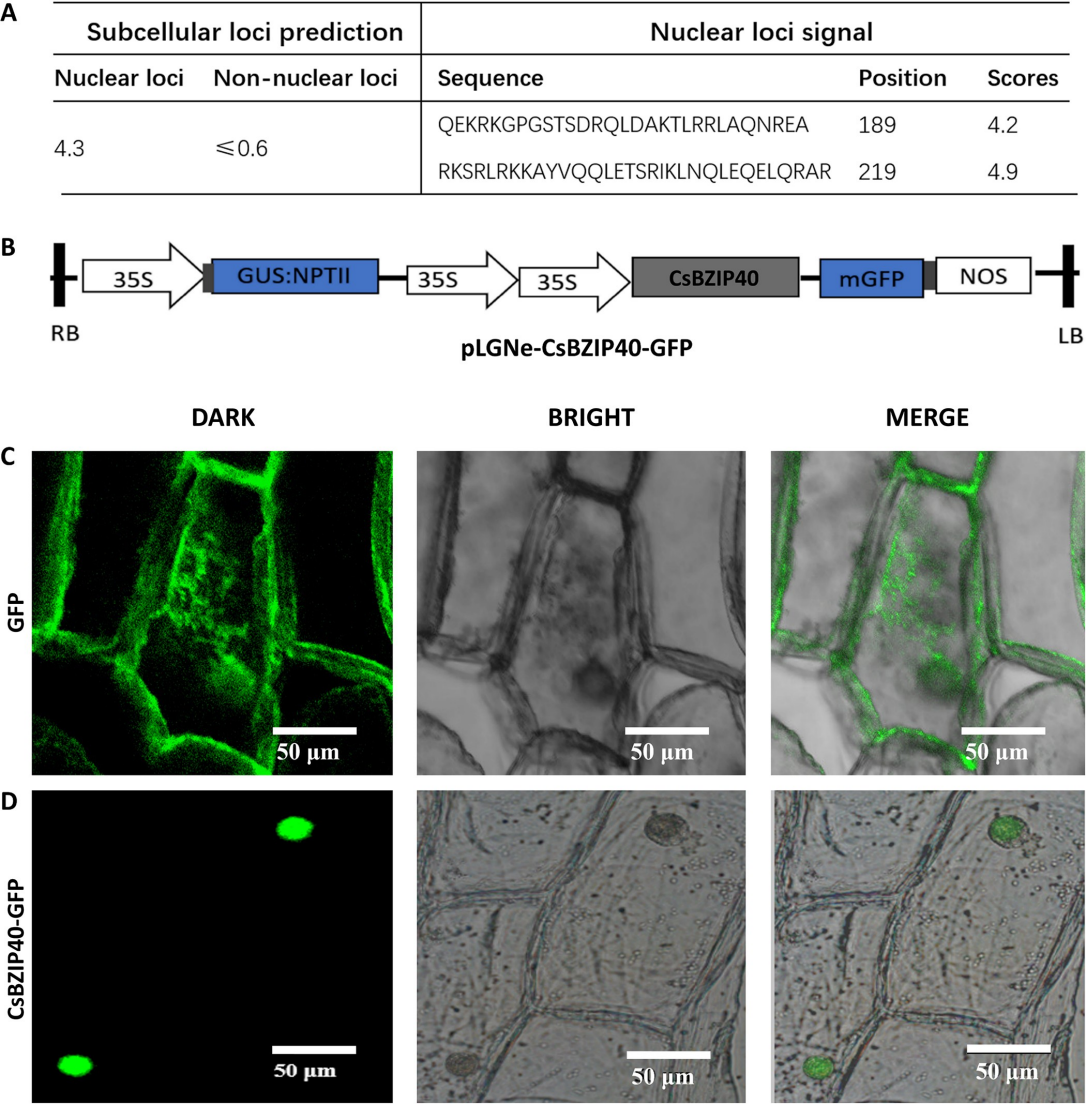
CsBZIP40 localizes to the nucleus. **(A)** Prediction of nuclear localization with CELLO and NLS. **(B)** Plasmid structure for transient expression to detect the subcellular localization of CsBZIP40. LB: left border, RB: right border. Fluorescent signals of GFP in onion epidermal cells **(C)** were used as the control. **(D)** The transient expression measured by CsBZIP40-GFP fluorescence. In C and D, scale bar represents 50 μm. Each field of view was observed in dark field (DARK), bright-field (BRIGHT) and merged imaging of bright and dark fields (MERGE).

Both software prediction and a transient expression system were used to ascertain the localization of CsBZIP40. CELLO indicated a nuclear loc value of 4.3 which was even larger than the non-nuclear loci value. NLS detected 2 nuclear localization signals in CsBZIP40, which suggested nucleic localization (Fig 2A). To validate the prediction, plasmid pLGNe-CsBZIP40-GFP containing CsBZIP40-GFP fusion protein controlled by a CMV35S promoter was constructed (Fig 2B). In onion epidermal cells infected with pLGNe-GFP, GFP transient expression was detected in both the cytoplasm and nucleus as determined by microscopic examination (Fig 2C), while cells containing the fusion vector displayed green fluorescence only in the nucleus (Fig 2D). Both prediction and transient expression indicate that CsBZIP40 is a nucleus localized protein which may play a role as a transcription factor to regulate the transcriptional expression of downstream genes.

### CsBZIP40 gene expression was induced by Xcc

**Fig 3:**
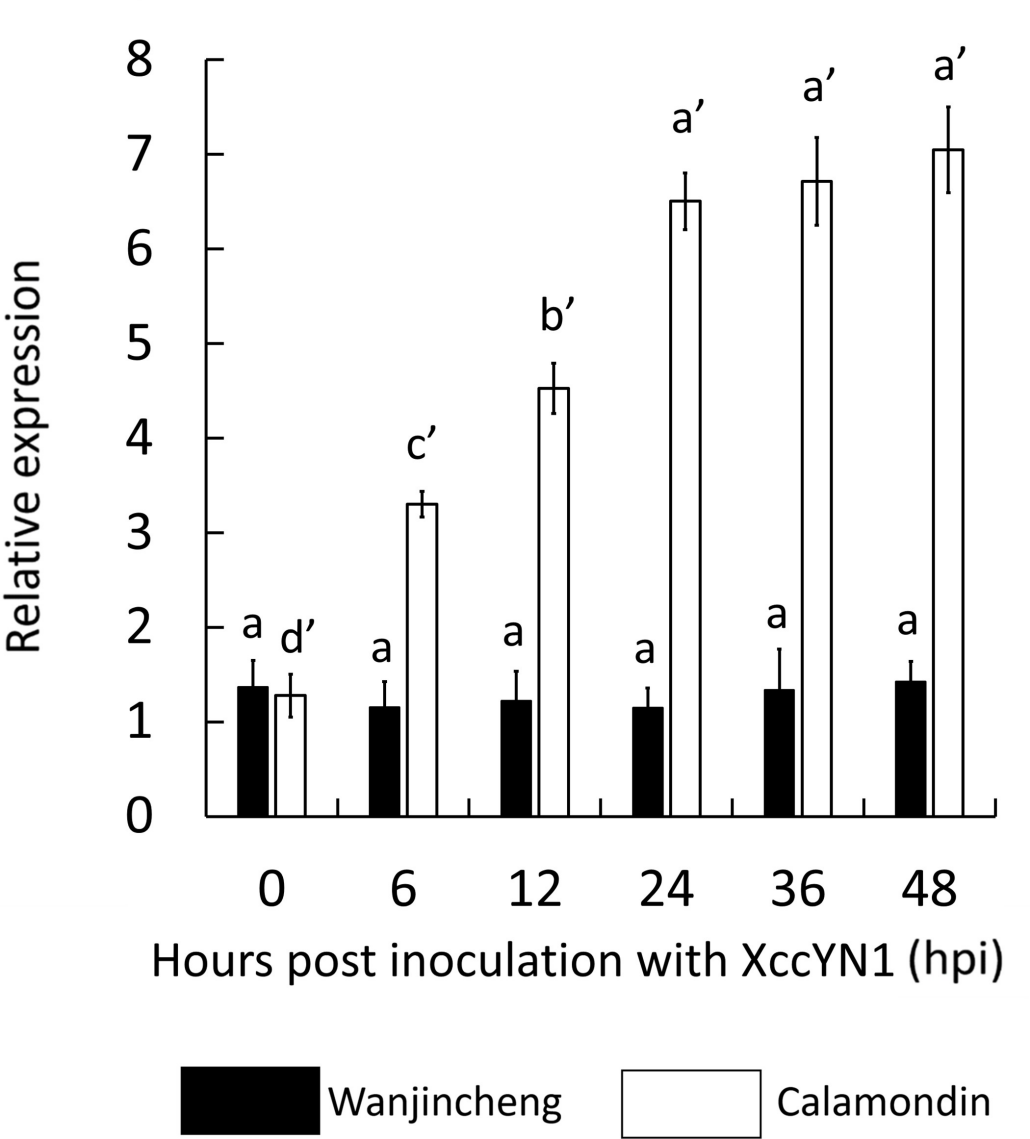
Relative expression of CsBZIP40 induced by Xcc. qRT-PCR was used for expression profiling induced by Xcc, and normalized using CsActin. Filled bars represent CsBZIP40 expression in Wanjincheng while empty bars represent expression in Calamondin. Samples collected at 0, 6, 12, 24, 36 and 48 hours post-inoculation (hpi) with XccYN1. LB medium was used as the control. The expression of all samples was compared with the control sample. Significant differences were detected (*: P < 0.05).

To analyze the relationship between CsBZIP40 expression and induction by Xcc, the expression patterns of CsBZIP40 at 0, 6, 12, 24, 36 and 48 hpi were measured by qRT-PCR in both canker sensitive Wanjincheng and canker resistant CalamondinThe results indicate that in Wanjincheng, there was no significant difference in CsBZIP40 expression after inoculation, while there was a significant increase in CsBZIP40 expression in Calamondin from 0 hpi and increasing to be maximal at 48 hpi (Fig 3). The expression of CsBZIP40 increased by approximately 7-fold compared with the minimum level. Expression profiles induced by Xcc indicated that CsBZIP40 might be a citrus canker related gene. In other words, increased expression of CsBZIP40 should be related to Xcc resistance.

### CsBZIP40 expression was induced by exogenous hormones

**Fig 4:**
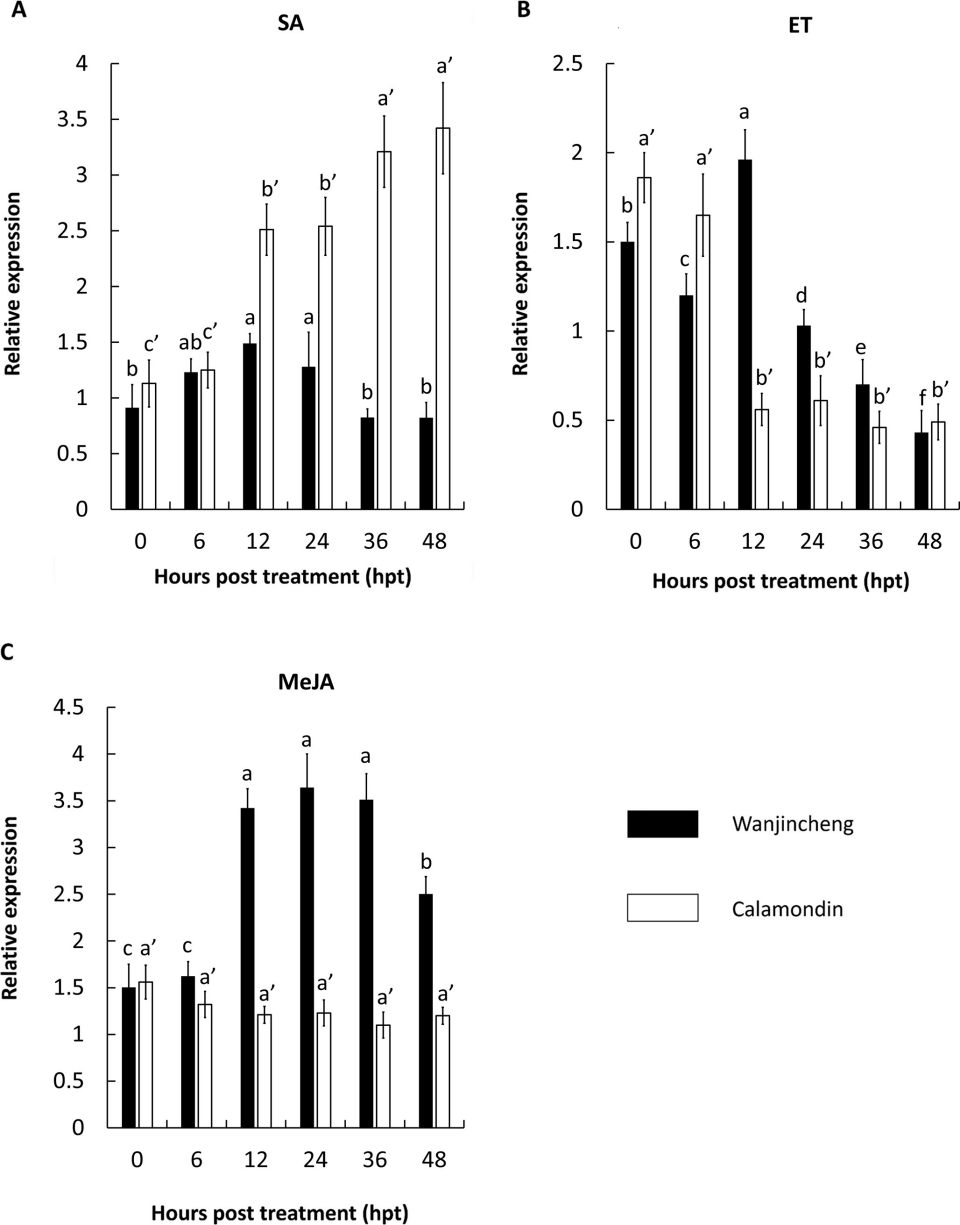
Relative expression of CsBZIP40 induced by different exogenous hormones. Expression profiles induced by SA induction **(A)**, MeJA induction **(B)** and ET induction **(C)** were quantified using qRT-PCR and normalized against CsActin. Filled bars represent Wanjincheng while empty bars represent Calamondin. Samples were collected at 0, 6, 12, 24, 36 and 48 hours post treatment (hpt). Control was sample inoculated with H_2_O_2_. Significant differences were detected (*: P < 0.05).

The overall expression of CsBZIP40 in Wanjincheng in response to exogenous SA did not elicit a significant change within the 48 hours of treatment except for a small increase at 12 hpt, while its expression in Calamondin increased from 12 hpt and was maintained at a high level 36 hpt. During these 48 hours, the expression of CsBZIP40 induced by Xcc infection increased by approximately 3-fold (Fig 4A). Ethylene (ET) induced significant changes in CsBZIP40 gene expression in both Calamondin and Wanjincheng. In Wanjincheng, CsBZIP40 was up-regulated by ET induction from 6 hpi but at 12 dpi, there was a sharp decline to a level even lower than that observed pre-infection. Expression in Calamondin always fell to a low level during the 48 hours’ treatment (Fig 4B). Conversely, the CsBZIP40 expression level in Calamondin induced by MeJA was not significantly different at any sampling time point while it increased significantly in Wanjincheng from 12 hpt and then decreased after 36 hpt (Fig 4C).

### Over-expression of CsBZIP40 conferred Xcc resistance to host plants

**Fig 5:**
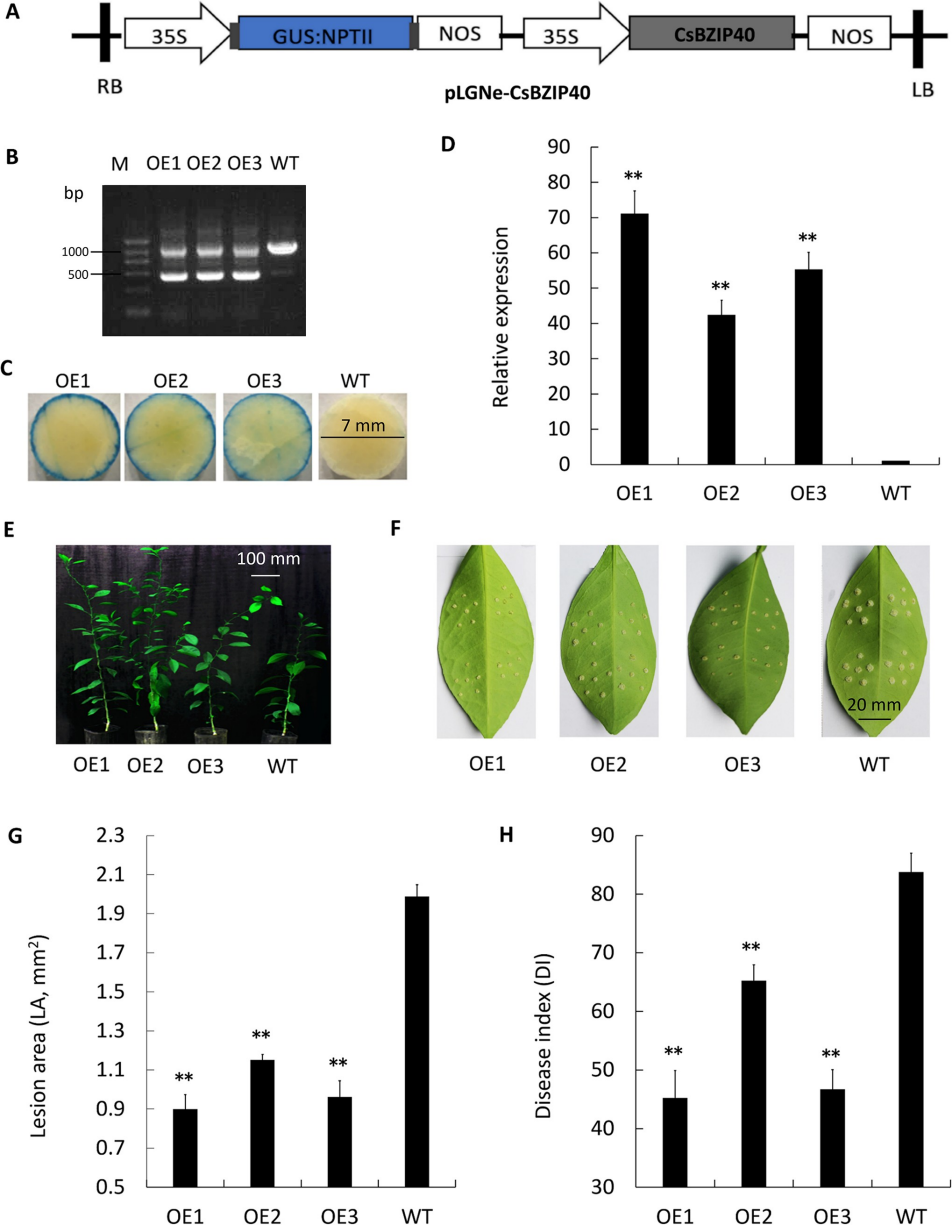
Over-expression of CsBZIP40 conferred Xcc resistance to transgenic citrus. **(A)** Structure of plasmid for over-expression assay. LB: left border, RB: right border. Transgenic lines were validated by **(B)** PCR and GUS stain (24 hours) of 7 mm leaf discs **(C)**. **(D)** Relative expression of CsBZIP40 in the over-expression transgenic lines quantified by qRT-PCR. **(E)** Phenotypes of over-expression lines. **(F)** Xcc-inoculated over-expression transgenic lines. Sampling and imaging at 10 dpi (days post inoculation). Lesion area (LA) **(G)** and disease index (DI) **(H)** of each transgenic line were investigated for resistance evaluation. WT: wild type; OE1 to OE3: over-expression transgenic lines. In (D), (G) and (H), a student’s t-test was used to calculate the significance of differences: (*: P < 0.05, **: P < 0.01). Error bars represent means ± SE.

Transgenic engineering of citrus aided the understanding of the role of CsBZIP40 and allowed further investigation of the regulatory mechanisms in Xcc resistance. In the case of over-expression, recombinant plasmid pLGNe-CsBZIP40 was constructed with CMV35S as the promoter and the GUS coding gene as a selection marker (Fig 5A). Three lines, denoted OE1 to OE3 with transgenic integration of CsBZIP40 were confirmed by PCR and GUS assays. By PCR, two characteristics electrophoresis bands were detected in OE positive lines while only one band was displayed in WT plants (Fig 5B). In the GUS assay, the blue staining on the edges of discs of leaves from positive lines was confirmation of a positive response compared to WT plants (Fig 5C). Transgenic lines OE1 to OE3 expressed a very high level of CsBZIP40 compared with the WT (70, 40 and 55-fold greater than WT, respectively) (Fig 5D). All 3 of the over-expressed lines exhibited normal growth similar to that of the WT (Fig 5E).

Inoculation with pinpricks was used to study resistance of transgenic lines OE1-OE3. At 10 dpi, the development of disease was examined. The size of lesions on over-expression transgenic lines were smaller than those on WT plants (Fig 5F), indicating that the pustules of Xcc infection had been inhibited by over-expression of CsBZIP40 while the greatest resistance was exhibited by OE1 followed by OE3 then OE2. Regarding lesion areas (LA), OE1 possessed the smallest lesions, like those of OE3, while OE2 possessed the largest (Fig 5G). Overall, lesion areas of the over-expression lines were only 45%-58% those of the WT. Disease index (DI), a measure of disease severity, was reduced by 22% (OE2)-45% (OE1) in the transgenic plants compared with the WT (Fig 5H). Combined, these results reveal that the over-expression lines had strong and stable resistance to citrus bacterial canker, line OE1 displaying the greatest resistance.

### RNAi silencing of CsBZIP40 led to susceptibility of the host to Xcc

**Fig 6:**
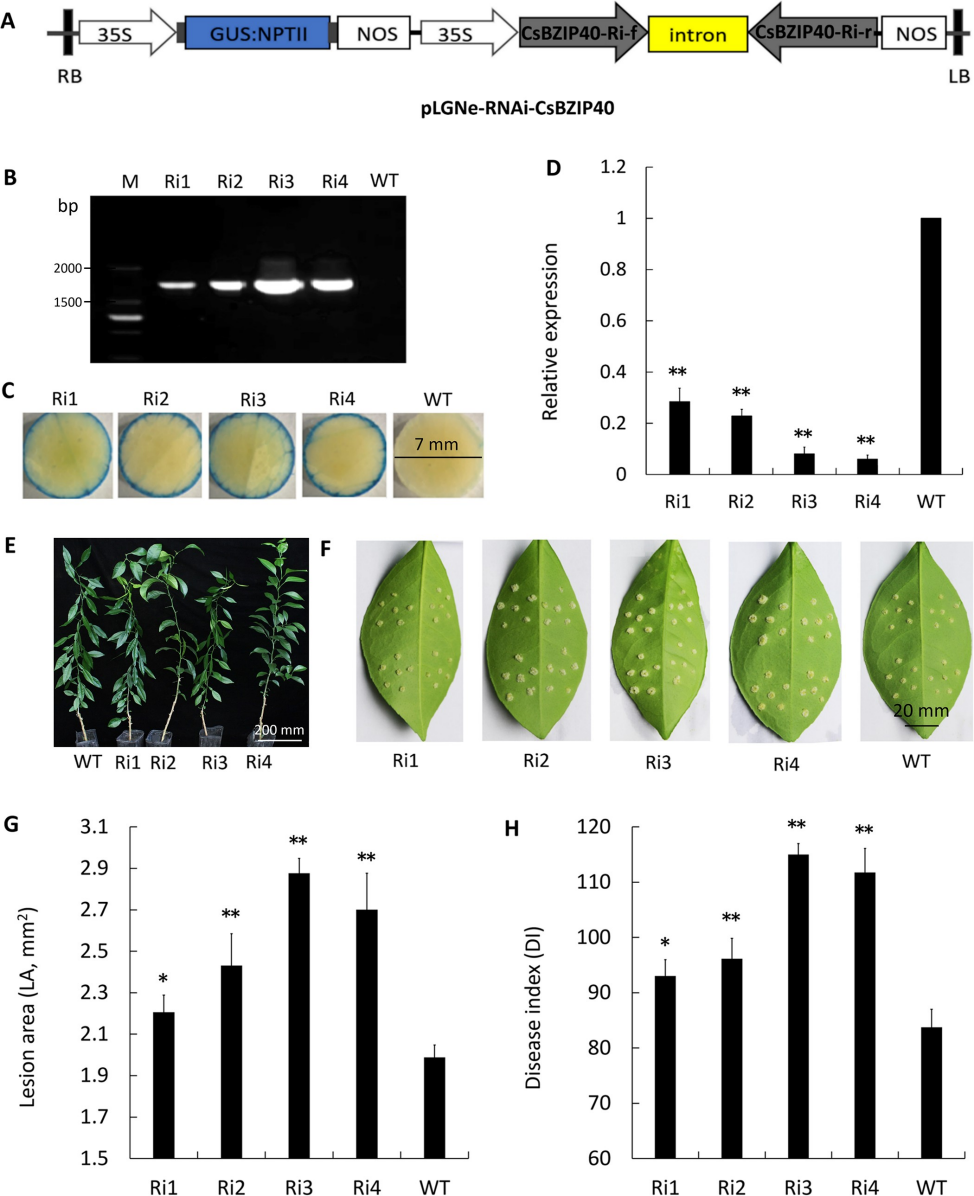
Silencing of CsBZIP40 in sweet orange led to Xcc susceptibility. **(A)** Structure of plasmid for RNAi assay. LB: left border, RB: right border. Transgenic lines were validated by **(B)** PCR and GUS stain (24 hours) of 7 mm leaf discs **(C)**. **(D)** Relative expression of CsBZIP40 in the RNAi transgenic lines quantified by qRT-PCR. **(E)** Phenotypes of RNAi transgenic lines. **(F)** Xcc-inoculated RNAi transgenic lines. Sampling and imaging at 10 dpi. Lesion area **(G)** and disease index **(H)** of each transgenic line was investigated for evaluation of resistance. WT: wild type; Ri1 to Ri4: RNAi transgenic lines. In (D), (G) and (H), a student’s t-test was used to calculate the significance of differences: (*: P < 0.05, **: P < 0.01). Error bars represent means ± SE.

To further elucidate the role of CsBZIP40, its expression was knocked down using an RNAi strategy (Fig 6A). Four RNAi lines (Ri1 to Ri4) were created in this study, validated by both PCR (Fig 6B) and GUS staining (Fig 6C). These four lines exhibited a relative low level of expression of CsBZIP40 compared with the WT, as demonstrated by qRT-PCR, especially Ri3 and Ri4 in which the transcriptional levels of CsBZIP40 were lower than 10% that of the WT (Fig 6D). The 4 RNAi-mutant lines also exhibited a normal growth rate which suggested that insertion of recombined RNAi fragments to the genome did not abnormally influence the plants (Fig 6E). In terms of CBC resistance, lines Ri1, Ri2, Ri3 and Ri4 exhibited an eruption of larger pustules compared with those of the WT (Fig 6F). Diseased lesions of the four RNAi-mutant lines were significantly physically larger than those of the WT, especially Ri3 and Ri4 (Fig 6G). Analysis of CBC severity revealed that the 4 RNAi-mutant lines had a significantly higher DI than the WT (Fig 6H). Consequently, disease severity increased by 11% (Ri1)-37% (Ri3). Summarizing the data above, we conclude that RNAi silencing of CsBZIP40 led to higher susceptibility to Xcc in these transgenic sweet orange plants.

### CsBZIP40 interacted directly with CsNPR1 and regulated the concentration of SA

**Fig 7:**
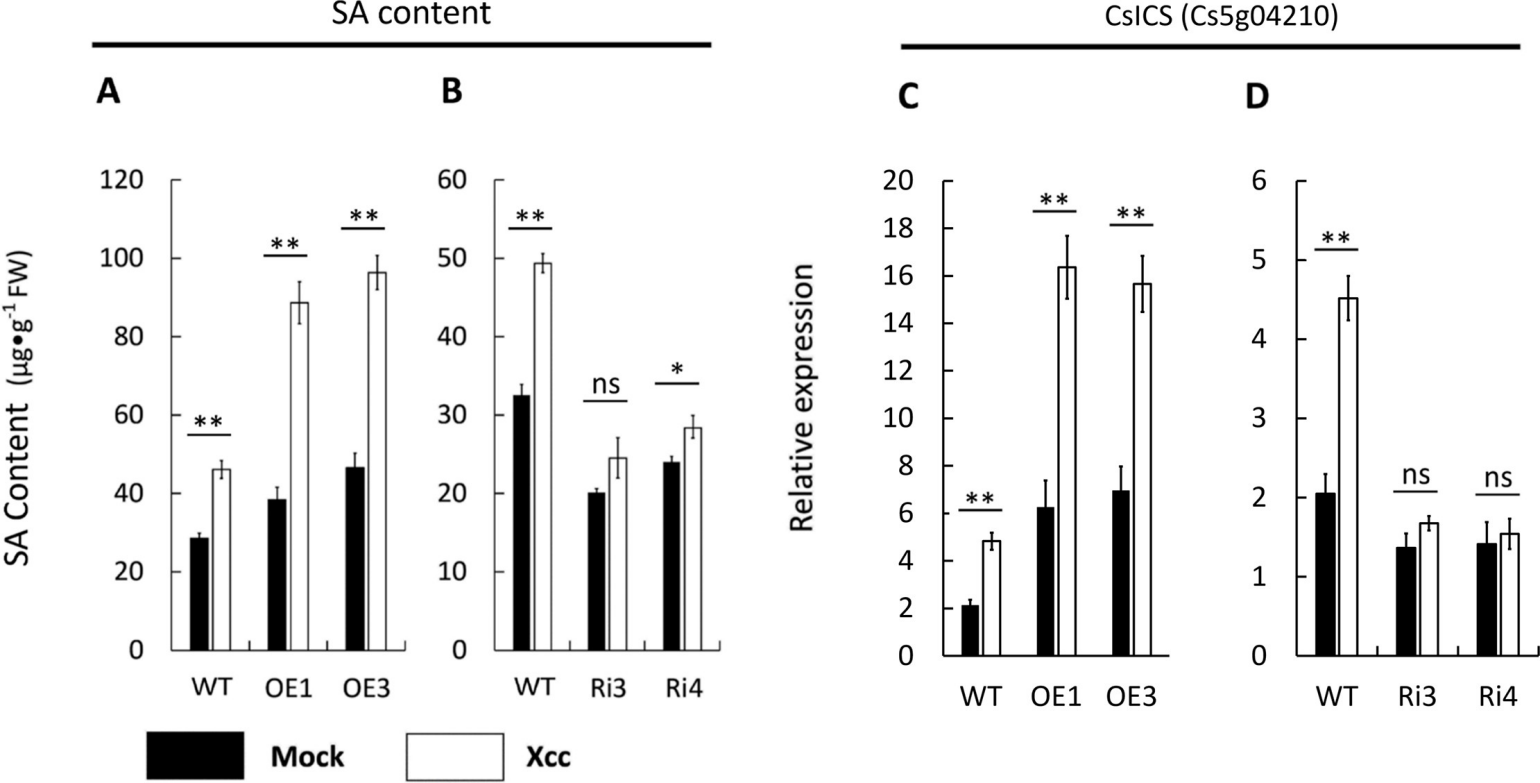
CsBZIP40 regulates the SA biosynthesis. The content of salicylic acid in CsBZIP40 over-expression lines **(A)** and RNAi lines **(B)** were measured with LB medium (Mock) (filled bars) and Xcc inoculation (empty bars) at 12 hpi. The expression profiles of CsICS **(C, D)** were quantified with LB medium (filled bars) and Xcc inoculation (empty bars) at 12 hpi by qRT-PCR in both over-expression and RNAi plants. A student’s t-test was used to calculate the significance of differences: (*: P < 0.05, **: P < 0.01, ns: no significance). Error bars represent means ± SE.

Taken together, these results suggest that CsBZIP40 might monitor the activity of Xcc and induce innate immunity to inhibit the process of infection by Xcc, thus reducing incidence of disease. BZIP TFs are known to function by interacting with other proteins. To obtain these CsBZIP40-interacting proteins during the response to Xcc infection, GST pull-down was conducted in this study. In total, 51 interacting proteins were pulled down (S5 Table) from OE1. From the interaction network constructed by STRING (https://string-db.org/) with *Arabidopsis thaliana* used as the reference, direct interaction between CsBZIP40 (ortholog of AtTGA9) and CsNPR1 (Cs4g14600) was predicted (S1 Fig). The NPR1 (non-expressor-of-pathogenesis-related-genes-1 protein) gene is a key regulator of the salicylic acid (SA)-mediated systemic acquired resistance (SAR) pathway [39]. This interaction between TGA and NPR1 has already been confirmed by several reports in other plant models [39–41]. The interaction of CsBZIP40 and CsNPR1 provided the possibility that CsBZIP40 was able to trigger the SA signaling pathway, leading to activation of pathogenesis-related (PR) genes. To verify the involvement of the SA signaling pathway triggered by CsBZIP40 in transgenic lines, the concentration of salicylic acid and expression of a number of SA-mediated genes were measured (Fig 7). The OE-lines with highest resistance (OE1 and OE3) and RNAi-lines with highest susceptibility to CBC (Ri3 and Ri4) were selected for phytohormone detection. Compared with WT lines, the SA content of OE1 and OE3 was significantly higher and up-regulated sharply by Xcc infection (Fig 7A) while in Ri3 and Ri4, the SA content was lower than the WT and insensitive to Xcc infection (Fig 7B). The relative expression of SA biosynthesis-related gene CsICS (CAP accession: Cs5g04210) was sensitively and sharply up-regulated relative to WT in CsBZIP40 over-expression plants upon Xcc infection while in RNAi lines, the expression of CsICS was down-regulated and not sensitive to the Xcc induction (Fig 7C and 7D). To summarize hormone content evaluation in transgenic lines before and after Xcc infection, we conclude that CsBZIP40 positively regulates SA synthesis.

### CsBZIP40 positively regulated SA-responding genes

**Fig 8:**
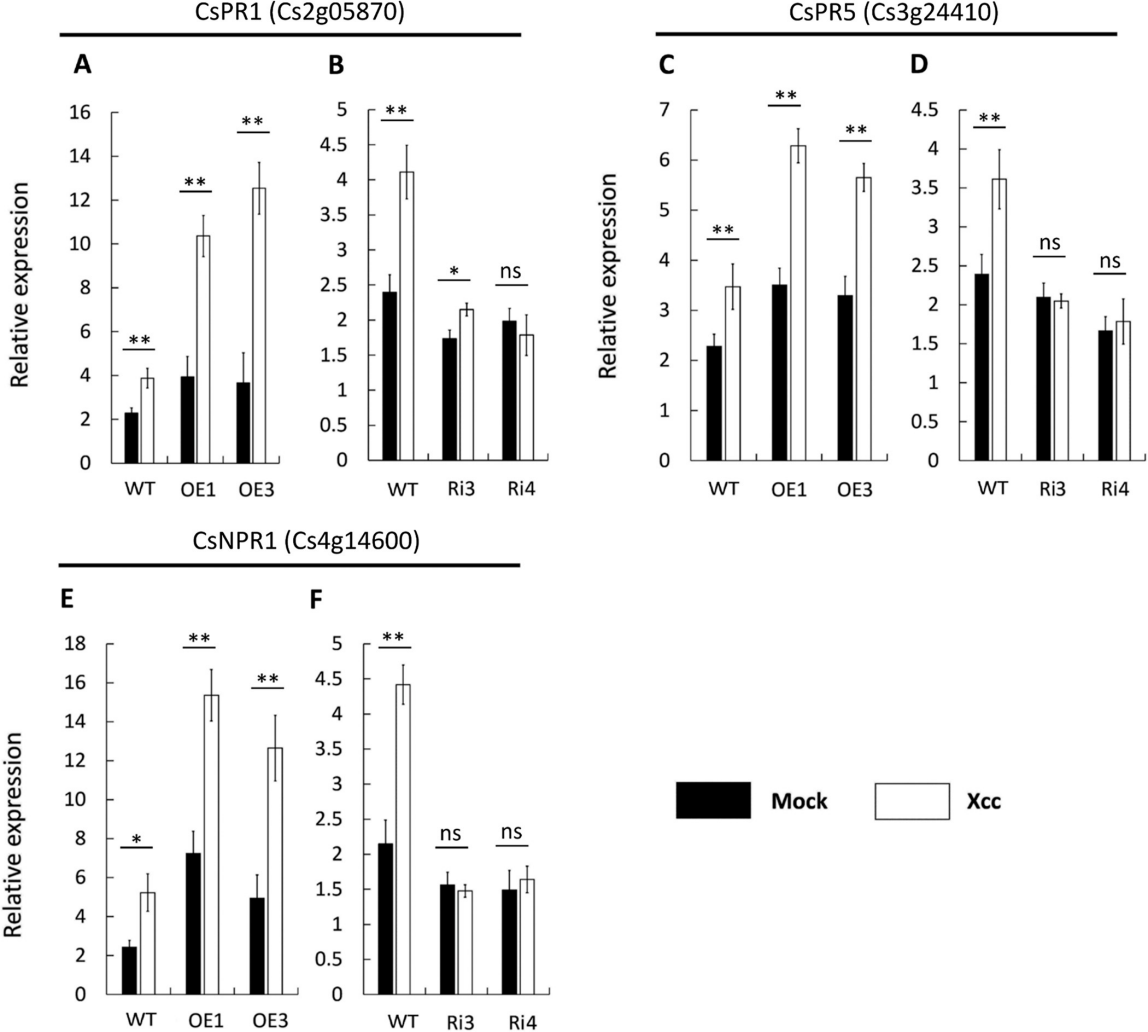
CsBZIP40 regulates CBC resistance through the SA pathway. The expression profiles of CsPR1 **(A, B)**, CsPR5 **(C, D)** and CsNPR1 **(E, F)** were quantified with LB medium (filled bars) and Xcc inoculation (empty bars) at 12 hpi by qRT-PCR in both over-expression and RNAi plants. A student’s t-test was used to calculate the significance of differences: (*: P < 0.05, **: P < 0.01, ns: no significance). Error bars represent means ± SE.

PR1 and PR5 have previously been confirmed as having involvement in the SA-responsive pathway [40]. To verify their involvement in the citrus SA pathway, expression profiles of the two PRs (CsPR1: Cs2g05870; CsPR5: Cs3g24410) were evaluated in the transgenic lines by qRT-PCR. As expected, the 2 genes were up-regulated and down-regulated in over-expression and RNAi lines, respectively (Fig 8A and 8B). Regarding Xcc infection, PR1 and PR5 were sensitively and sharply up-regulated by Xcc induction in CsBZIP40 over-expression lines (Fig 8A and 8C) while in RNAi lines, compared to WT, the expression of PR1 and PR5 was not sensitive to Xcc induction (Fig 8B and 8D). NPR1 which interacted with CsBZIP40 was also evaluated in this study. Consistent with the PR genes and SA content, the expression of CsNPR1 was also differentially expressed in transgenic lines compared with WT plants (Fig 8E and 8F). This suggests that the expression level of CsNPR1 was also regulated by CsBZIP40 expression.

### The model of CsBZIP40 demonstrated enhanced CBC resistance via the SA signaling pathway

**Fig 9:**
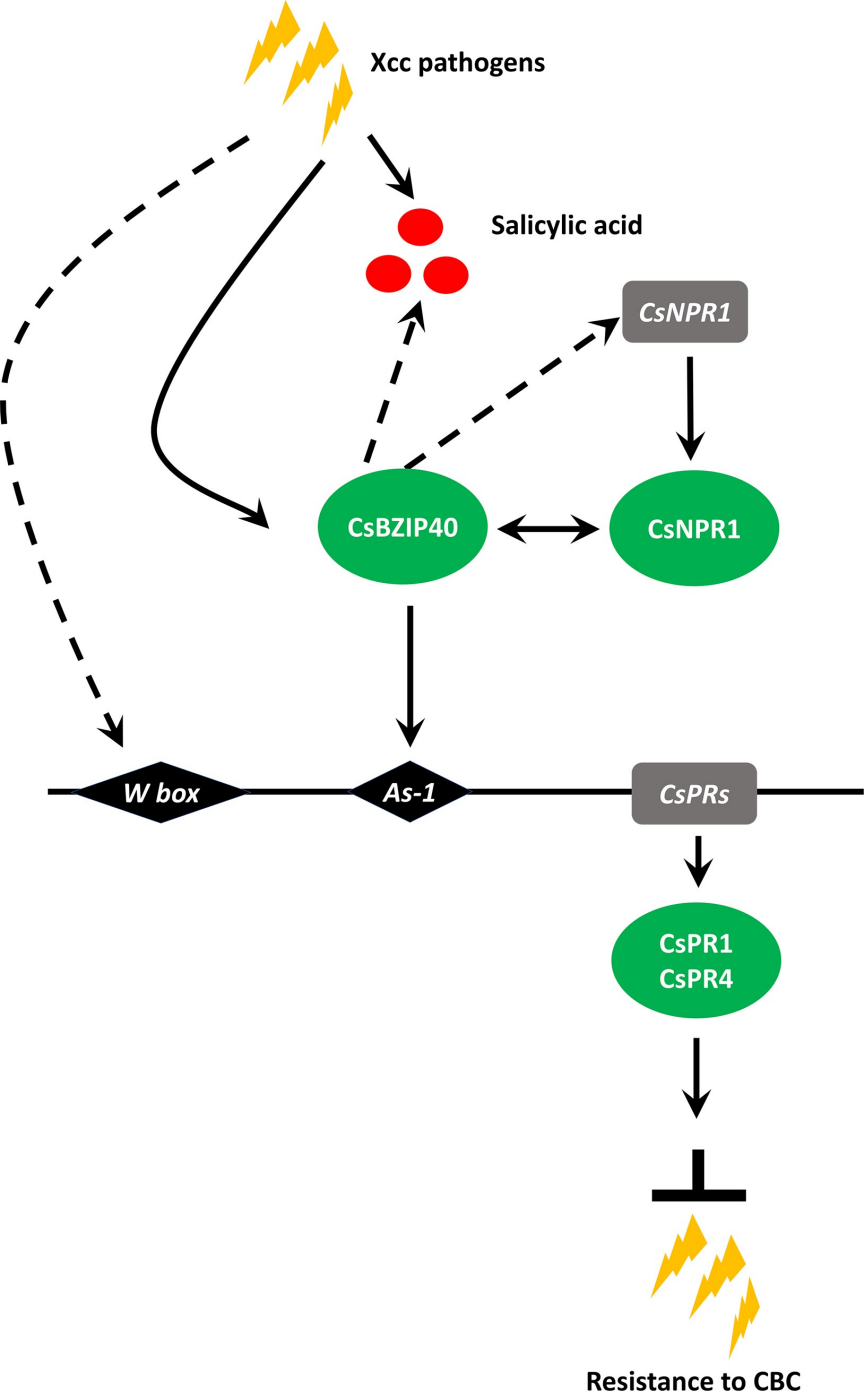
Modulation of CsBZIP40 in CBC resistance enhancement via the SA signaling pathway. Xcc induces the expression of CsBZIP40 and salicylic acid synthesis. Through the interaction of CsNPR1, CsBZIP40 activates the expression of PRs as a transcriptional regulator. The PRs regulate the resistance to CBC. Red cycles: salicylic acid; gray rectangles: genes involved in the modulation model; green ellipses: proteins involved in the modulation model; black diamonds: elements in the promotor; arrows: regulation; two—headed arrow: protein interaction; dashed line: process not yet clear.

CsBZIP40 over-expression strains and plants silenced with RNAi exhibited regulation of the SA signaling pathway combined with positive improvement in CBC resistance. In a CBC resistance variety, Xcc inoculation induced expression of CsBZIP40 and salicylic acid synthesis. CsBZIP40 interaction with CsNPR1 activated the expression of PRs as a transcriptional regulator (Fig 9). PRs regulate resistance to CBC. This model revealed the reason over-expression of CsBZIP40 can improve CBC resistance in a CBC susceptible variety.

## Discussion

### CsBZIP40 is a candidate CBC resistance gene influenced by Xcc infection

Basic-region leucine zipper (bZIP) transcription factors regulate numerous responses in plants, such as pathogen defense and stress signaling, *etc*. [42, 43]. BZIPs are proteins containing a basic domain involved in transcriptional regulation. BZIPs belong to a multigenic family which has been comprehensively identified or predicted in a number of plant genomes. In this study, from the sweet orange genome, 47 BZIPs (S2 Table) were annotated, fewer than in Arabidopsis (75) [12], maize (*Zea mays*, 125) [7], soybean (*Glycine max*, 131) [44] or rice (*Oryza sativa*, 89) [11]. Based on phylogeny, CsBZIP40 is an orthologous gene of AtTGA in Arabidopsis which is clustered in group D and involved in pathogen defense, as reported previously [39, 41, 45, 46]. Interestingly, in the present study, CsBZIP40 can be differentially induced by Xcc infection in CBC-resistant and susceptible citrus species (Fig 3). This finding provided insight into the involvement of CsBZIP40 in the defense against Xcc. In other words, CsBZIP40 is possibly a CBC-related gene. There are examples in the functional study of BZIPs involved in bio-stress. In pepper, CABZIP1, a pathogen-induced BZIP transcription factor, has been shown to confer strong resistance to pathogen infection [47]. Tobacco BZIP transcription factor TGA2.2 and related factor TGA2.1 have plant defense response and plant development functionality [48]. To our knowledge, few CsBZIPs have been identified or characterized in citrus, and no detailed functional studies of CsBZIPs have been reported in biotic stress, especially in CBC tolerance.

### CsBZIP40 regulates CBC resistance by the SA pathway in the presence of NPR1

The role of positive regulation of CsBZIP40 was validated in this study by over and under expression. Over-expression plants demonstrated Xcc resistance while silenced plants were more susceptible to Xcc infection (Figs 5 and 6). These findings conferred positive regulation of CsBZIP40 to CBC tolerance. Our results suggest that an interaction existed between CsBZIP40 and CsNPR1 (S1 Fig). Previous studies have reported that TGA (BZIPs in Arabidopsis) is able to transcriptionally activate pathogenesis-related (PR) genes through use of a SA-regulated mechanism at primary infection sites, and subsequently in distant regions. In the DNA binding and transactivation processes, NPR1 is always required and physically interact with TGAs [39, 49]. In the presence of SA, NPR1 disulfide bonds are hydrolyzed by modification of the intracellular redox, when monomeric NPR1 from the oligomeric complex is released. NPR1 monomers translocate into the nucleus to interact with TGA transcription factors to form a transcriptionally-active subcomplex. which activates the expression of pathogenesis-related (PR) genes [50, 51]. It has been shown that in the nucleus of SA-treated cells of *Arabidopsis thaliana*, TGA2 and NPR1 are able to activate gene expression in transiently transformed *Arabidopsis thaliana*, whereas TGA2 alone cannot [52]. This study further characterized the precise mechanisms of SAR induction and functions of the NPR1-TGA-DNA complex. In the present study, CsBZIP40 possibly functioned like AtTGA2 in Arabidopsis. CsBZIP40 was found to positively regulate CsNPR1 and SA content which has not been demonstrated in other studies. The mechanism remains unclear and requires additional study.

## Conclusions

In conclusion, the role of CsBZIP40, a group-D BZIP transcription factor, in canker resistance was analyzed, demonstrating that it localized in the nucleus and could be clearly induced by hormones and Xcc infection. Over-expression of CsBZIP40 conferred Xcc resistance while RNAi silencing led to Xcc susceptibility. When interacting with NPR1, CsBZIP40 could up-regulate SA production and activate genes involved in the SA signaling pathway to further activate PR1 which conferred Xcc resistance to the host. This study provided the possibility of improving CBC resistance by over-expression of CsBZIPs.

## Supporting information

S1 Fig Visualization of the interaction network of proteins obtained from GST pull-down. The network was constructed using the STRING database with a score of 0.4 and Arabidopsis thaliana as the reference. Network nodes represent proteins with 3D structure either known or predicted. Lines between nodes represent known protein-protein interactions. The colors of lines represent the data source. TGA9 was the ortholog gene of CsBZIP40.
(DOCX)

S1 Table qRT-PCR Primers for a number of genes in this study. Primers were designed with NCBI Primer Blast software (https://www.ncbi.nlm.nih.gov/tools/primer-blast/index.cgi).
(XLSX)

S2 Table List and detailed information of all BZIPs in *Citrus sinensis*. MW: molecular weight. AA.: amino acid. CHR: chromosome. PI: isoelectric point. Link to Phytozome: https://phytozome.jgi.doe.gov/pz/ portal.html. Link to CAP: http://citrus.hzau.edu.cn/orange/index.php.
(XLSX)

S3 Table Distribution ratio of CsBZIP genes among chromosomes. Sizes of chromosomes were obtained from CAP (http://citrus.hzau.edu.cn/orange/index.php).
(XLSX)

S4 Table List of BZIPs in *Arabidopsis thaliana* for phylogenetic analysis. All data were annotated from Phytozome (https://phytozome.jgi.doe.gov/).
(XLSX)

S5 Table List of proteins interacting with CsBZIP40 during Xcc infection. Listed genes were obtained from GST pull-down, identified by LC/MS and annotated based on the CAP database (http://citrus.hzau.edu.cn/orange/index.php).
(XLSX)
