# Supplementary material for: CsBZIP40, a BZIP transcription factor in sweet orange, plays a positive regulatory role in citrus bacterial canker response and tolerance
Source: PLoS One. 2019 Oct 4;14(10):e0223498. doi: 10.1371/journal.pone.0223498 (PMC6777757; doi:10.1371/journal.pone.0223498)

**S1 Fig. Visualization of the interaction network of proteins obtained from GST pull-down.** The network was constructed using STRING database with 0.4 as a score and with *Arabidopsis thaliana* as the reference. Network nodes represent proteins with 3D structure is known or predicted. Lines between nodes represent known protein-protein interactions. Different colors of lines represent different data source. TGA9 was the ortholog gene of CsBZIP40.


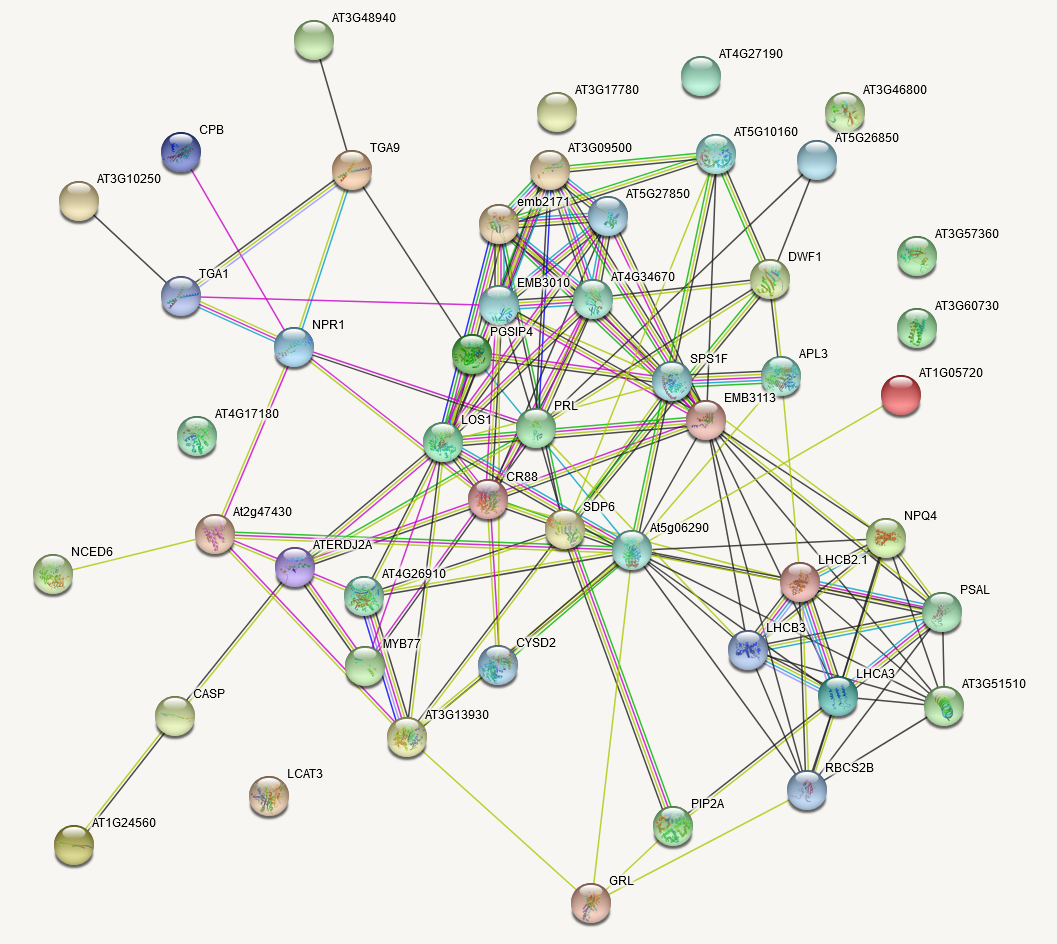

Supplement: S1 Fig — The network was constructed using the STRING database with a score of 0.4 and Arabidopsis thaliana as the reference. Network nodes represent proteins with 3D structure either known or predicted. Lines between nodes represent known protein-protein interactions. The colors of lines represent the data source. TGA9 was the ortholog gene of CsBZIP40. (DOCX) [file pone.0223498.s001.docx]
